# Supplementary material for: Uncovering a conserved vulnerability site in SARS‐CoV‐2 by a human antibody
Source: EMBO Mol Med. 2021 Nov 17;13(12):e14544. doi: 10.15252/emmm.202114544 (PMC8646660; doi:10.15252/emmm.202114544)
Supplement: Supplementary file 1 — Appendix [file EMMM-13-e14544-s003.pdf]

Uncovering a conserved vulnerability site in SARS-CoV-2 by a human antibody

Authors: Tingting Li[1,2,#], Hongmin Cai[1,2,#], Yapei Zhao[2,3,#], Yanfang Li[1,2,#], Yanling Lai[1,2,#], Hebang Yao[1,2,#], Liu Daisy Liu[1,2,#], Zhou Sun[4,#], Martje Fentener van Vlissingen[5,6], Thijs Kuiken[6,7], Corine H. GeurtsvanKessel[6,7], Ning Zhang[8], Bingjie Zhou[2,3], Lu Lu[2,3], Yuhuan Gong[2,8], Wenming Qin[9], Moumita Mondal[3,10], Bowen Duan[2,3], Shiqi Xu[2,3], Audrey S Richard[6], Hervé Raoul[6], Jianfeng Chen[1], Chenqi Xu[1], Ligang Wu[1], Haisheng Zhou[1,2], Zhong Huang[3,10], Xuechao Zhang[4], Jun Li[4], Yanyan Wang[1,2], Yuhai Bi[2,8], Barry Rockx[6,7], Junfang Chen[4,*], Fei-Long Meng[1,2,*], Dimitri Lavillette[3,10,11,*], Dianfan Li[1,*]

Table of Contents

Appendix Table S1 – S2

Appendix Fig S1 – S4

Full wwPDB X-ray Structure Validation Report

Appendix Table S1. Amino-acid sequence of neutralizing antibodies identified in this study.

scFv	Sequence
scFD20	QVQLQQSGAEVKKPGSSVKVSCKASGGTFSSYAFSWVRQAPGQGLEWMGRIIPILGIANYAQKF<br>QGRVTITADISTSTAYMALSSLRSEDTAVYYCAKVEVNIGMDDYYYYYGMDVWGQGTTVTVSS<br>GGGSGGGGSGGGGSSYVLTQPPSVSVSPGQTARITCSGDALPKQYAYWYQQKPGQAPVLVIYKD<br>SERPSGIPERFSGSSSGTTVTLTISGVQAEDEADYYCQSADSSGTYVVFGGGTKLTVL
4LC11	QVQLVQSGTEVKKPGASVKVSCKASGYTFTSYYMHWVRQAPGQGLEWMGIINPGGGSTSYAQ<br>KFQGRVTMTTDTSTSTVYMELSSLRSEDTAMYYCARDLYGVPAVGGMDVWGQGTLVTVSSGG<br>GGSGGGGSGSGGSQSALTQPPSASGTPGQRVTISCSGSSSNIGSNSVNWYQQLPGTAPKLLIYGSS<br>QRPSGVPARFSGSKSGTSASLAISGLRSDDEAYYYCAAWDDSLGGAVFGGGTQLTVL
4KB2	EVQLVQSGAEVTQPGASVKVSCKASGYTFTSYYMHWVRQAPGQGLEWMGIINPGGGSTSYAQ<br>KFQGRVTMTTDTSTSTVYMELSSLRSEDTAMYYCARDLYGVPAVGGMDVWGQGTLVTVSSGG<br>GGSGGGGSGGGGSETTLTQSPATLSVSPGERATLSCRASQTVGGNFLAWYQQKPGRAPRLLIYG<br>TSGRAAGIPDRFTGSASGTDFTLTISRLDPEDFAVYYCQQRSKWYTFGQGTKLEIK
4KC9	QVQLVQSGTEVKKPGASVKVSCKASGYTFTSYYMHWVRQAPGQGLEWMGIINPGGGSTSYAQ<br>KFQGRVTMTTDTSTSTVYMELSSLRSEDTAMYYCARDLYGVPAVGGMDVWGQGTTVTVSSGG<br>GGSGGGGSGGGGPDIQMTQSPSSLSASVGDRVTITCRASQSISSYLNWYQQRPGKAPKLLIYAAS<br>SLQSGVPSRFSGTTSGTDFTLTISSLQPEDFATYYCQQANSFPLTFGGGTKLEIK
4KC12	QVQLVQSGTEVKKPGASVKVSCKASGYTFTSYYMHWVRQAPGQGLEWMGIINPGGGSTSYAQ<br>KFQGRVTMTTDTSTSTVYMELSSLRSEDTAMYYCARDLYGVPAVGGMDVWGQGTTVTVSSGG<br>GGSGGGGSGGGGSDIQMTQSPSSVSASVGDRVTITCRASQYISSRFAWYQQQPGKAPKLLIYAAS<br>TLQSGVPSRFSGTTSGTDFTLTISSLQPEDFATYYCQQASSFPLTFGGGTKVEIK
4KD8	QVQLVQSGAEVKKPGASVKVSCKASGYTFTSYYMHWVRQAPGQGLEWMGIINPGGGSTSYAQ<br>KFQGRVTMTTDTSTSTVYMELSSLRSEDTAMYYCARDLYGVPAVGGMDVWGQGTLVTVSSGG<br>GGSGGGGSGGGGSEIVLTQSPATLSLSPGERATLSCRASQSVSSYLAWYQQKPGQAPRLLIYDAS<br>NRATGIPARFSGSGSGTDFTLTISSLEPEDFAVYYCQQRSNWPRVITFGQGTRLEIK
4KE8	QVQLVQSGTEVKKPGASVKVSCKASGYTFTSYYMHWVRQAPGQGLEWMGIINPGGGSTSYAQ<br>KFQGRVTMTTDTSTSTVYMELSSLRSEDTAMYYCARDLYGVPAVGGMDVWGQGTLVTVSSGG<br>GGSGGGGSGSGGSDIQMTQSPSSLSASVGDRVTITCRASQNIGIYLNWYQQKPGKAPELLLYTAS<br>TLQSGVPSRFRGSGSGTDFTLTISSLQHEDFATYYCQQSYTTPPTFGGGTKVDIK

Appendix Table S2. Primers used in the construction of scFv phage display libraries.

Name[a]	Sequence (5’-3’)[b]
**IgH 1st PCR**	
5' L-VH1	**ACAGGTGCCCACTCCCAGGTGCAG**
5' L-VH3	**AAGGTGTCCAGTGTGARGTGCAG**
5' L-VH4/6	**CCCAGATGGGTCCTGTCCCAGGTGCAG**
5' L-VH5	**CAAGGAGTCTGTTCCGAGGTGCAG**
3' anti-Cγ CH1	**TCTTGTCCACCTTGGTGTTGCT**
**IgH 2nd PCR**	
5' phage-all-VH1	GCCGGCCATGGCAGCCGGCTCTTCA**CAGGTGCAGCTGGTGCAGTCTGG**
5' phage-all-VH6-1	GCCGGCCATGGCAGCCGGCTCTTCA**CAGGTACAGCTGCAGCAGTCAGG**
5' phage-all-VH2	GCCGGCCATGGCAGCCGGCTCTTCA**CAGGTCACCTTGARGGAGTCTGG**
5' phage-all-VH3	GCCGGCCATGGCAGCCGGCTCTTCA**GAGGTGCAGCTGGTGGAGTCTGG**
5' phage-all-VH4	GCCGGCCATGGCAGCCGGCTCTTCA**CAGGTGCAGCTGCAGGAGTCGGG**
5' phage-all-VH5	GCCGGCCATGGCAGCCGGCTCTTCA**GAGGTGCAGCTGTTGCAGTCTG**
3' GS JH1/2 /4/5	GAGCCACCTCCGCCGCTACCGCCGCCTCCAGA**TGAGGAGACGGTGACCAG**
3' GS JH3	GAGCCACCTCCGCCGCTACCGCCGCCTCCAGA**TGAAGAGACGGTGACCATTG**
3' GS JH6	GAGCCACCTCCGCCGCTACCGCCGCCTCCAGA**TGAGGAGACGGTGACCGTG**
**Igλ 1st PCR**	
5′ L Vλ 1	**GGTCCTGGGCCCAGTCTGTGCTG**
5′ L Vλ 2	**GGTCCTGGGCCCAGTCTGCCCTG**
5′ L Vλ 3	**GCTCTGTGACCTCCTATGAGCTG**
5′ L Vλ 4/5	**GGTCTCTCTCSCAGCYTGTGCTG**
5′ L Vλ 6	**GTTCTTGGGCCAATTTTATGCTG**
5′ L Vλ 7	**GGTCCAATTCYCAGGCTGTGGTG**
5′ L Vλ 8	**GAGTGGATTCTCAGACTGTGGTG**
3' C λ	**CACCAGTGTGGCCTTGTTGGCTTG**
**Igλ 2nd PCR**	
5' GSI Vλ1	AGCGGCGGAGGTGGCTCAGGCGGTGGCGGAAGT**CAGTCTGTGCTGACKCAG**
5' GSVλ2	AGCGGCGGAGGTGGCTCAGGCGGTGGCGGAAGT**CAGTCTGCCCTGACTCAG**
5' GS Vλ3	AGCGGCGGAGGTGGCTCAGGCGGTGGCGGAAGT**TCCTATGAGCTGACWCAG**
5' GS Vλ4/5	AGCGGCGGAGGTGGCTCAGGCGGTGGCGGAAGT**CAGCYTGTGCTGACTCA**
5' GS Vλ6	AGCGGCGGAGGTGGCTCAGGCGGTGGCGGAAGT**AATTTTATGCTGACTCAG**
5' GS Vλ7/8	AGCGGCGGAGGTGGCTCAGGCGGTGGCGGAAGT**CAGRCTGTGGTGACYCAG**
3' phage-His-Jλ -1	GATGGTGATGGTGGTGAGCTCTTCC**TAGGACGGTGACCTTGGTCCC**
3' phage-His-Jλ -2	GATGGTGATGGTGGTGAGCTCTTCC**TAGGACGGTCAGCTTGGTCCC**
3' phage-His-Jλ -3	GATGGTGATGGTGGTGAGCTCTTCC**GAGGACGGTCAGCTGGGTGCC**
**Igκ 1st PCR**	
5’ L Vκ1/2	**ATGAGGSTCCCYGCTCAGCTGCTGG**
5’ L Vκ3	**CTCTTCCTCCTGCTACTCTGGCTCCCAG**
5’ L Vκ4	**ATTTCTCTGTTGCTCTGGATCTCTG**
3’ C κ 543	**GTTTCTCGTAGTCTGCTTTGCTCA**
**Igκ 2nd PCR**	
5’ GS-all-Vκ1	AGCGGCGGCGGCGGCTCTGGTGGTGGTGGATCC**GACATCCAGATGACCCAGTCTCC**
5’ GS –all-Vκ6	AGCGGCGGCGGCGGCTCTGGTGGTGGTGGATCC**GAAATTGTGCTGACTCAGTCTCC**
5’ GS-all-Vκ2	AGCGGCGGCGGCGGCTCTGGTGGTGGTGGATCC**GATGTTGTGATGACTCAGTCTCC**
5’ GS-all-Vκ3	AGCGGCGGCGGCGGCTCTGGTGGTGGTGGATCC**GAAATTGTGTTGACGCAGTCTCC**
5’ GS-all-Vκ4	AGCGGCGGCGGCGGCTCTGGTGGTGGTGGATCC**GACATCGTGATGACCCAGTCTCC**
5’ GS-all-Vκ5	AGCGGCGGCGGCGGCTCTGGTGGTGGTGGATCC**GAAACGACACTCACGCAGTCTCC**
3’ phage-His-Jκ1	GATGGTGATGGTGGTGAGCTCTTCC**TTTGATTTCCACCTTGGTCCC**
3’ phage-His-Jκ2	GATGGTGATGGTGGTGAGCTCTTCC**TTTGATCTCCAGCTTGGTCCC**
3’ phage-His-Jκ3	GATGGTGATGGTGGTGAGCTCTTCC**TTTGATATCCACTTTGGTCCC**
3’ phage-His-Jκ4	GATGGTGATGGTGGTGAGCTCTTCC**TTTGATCTCCACCTTGGTCCC**
3’ phage-His-Jκ5	GATGGTGATGGTGGTGAGCTCTTCC**TTTAATCTCCAGTCGTGTCCC**

[a]Sense primers start with 5’ and antisense primers start with 3’. [b]bold letters denote nucleotide sequences annealing to germline-specific regions and plain letters denote those for Gibson cloning.

A

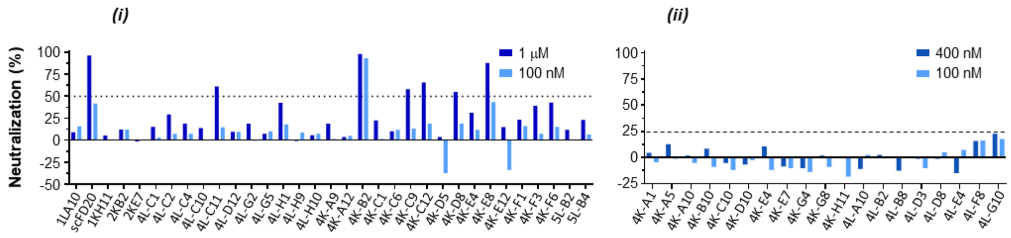

B

```
         10         20         30         40         50         60         70
QVQLQQSGAE  VKKPGSSVKV  SCKASGGTFS  SYAFSWVRQA  PGQGLEWMGR  IIPILGIANY AQKFQGRVTI
                              H-CDR1                          H-CDRH2
         80         90         100        110        120       127
TADISTSTAY  MALSSLRSED  TAVYYCAKVE  VNIGMDDYYY  YYGMDVWGQG  TTVTVSS GGGSGGGGSGGGGS
                                       H-CDR3
         10'        20'        30'        40'        50'        60'        70'
SYVLTQPPSV  SVSPGQTARI  TCSGDALPKQ  YAYWYQQKPG  QAPVLVIYKD  SERPSGIPER FSGSSSGTTV
                             L-CDR1                  L-CDR2
         80'        90'        100'
TLTISGVQAE  DEADYYCQSA  DSSGTYVVFG  GGTKLTVL
                   L-CDR3
```

Appendix Fig S1. Identification of neutralizing antibodies.

(**A**) Fifty-three scFv antibodies that bind to RBD on gel filtration were subjected to neutralization assays using SARS-CoV-2 pp and scFv at indicated concentrations.

(**B**) Amino-acid sequence of scFD20. Complementarity-determining regions (CDRs) from the heavy chain (H-CDRs) and the light chain (L-CDRs) are underlined. A prime symbol denotes residues from the light chain. The mature protein contained amino acids GSSSQ at the N-terminus and SAGRAGEQKLISEEDLNSAVDHHHHHH at the C-terminus for biotinylation and affinity purification purposes.

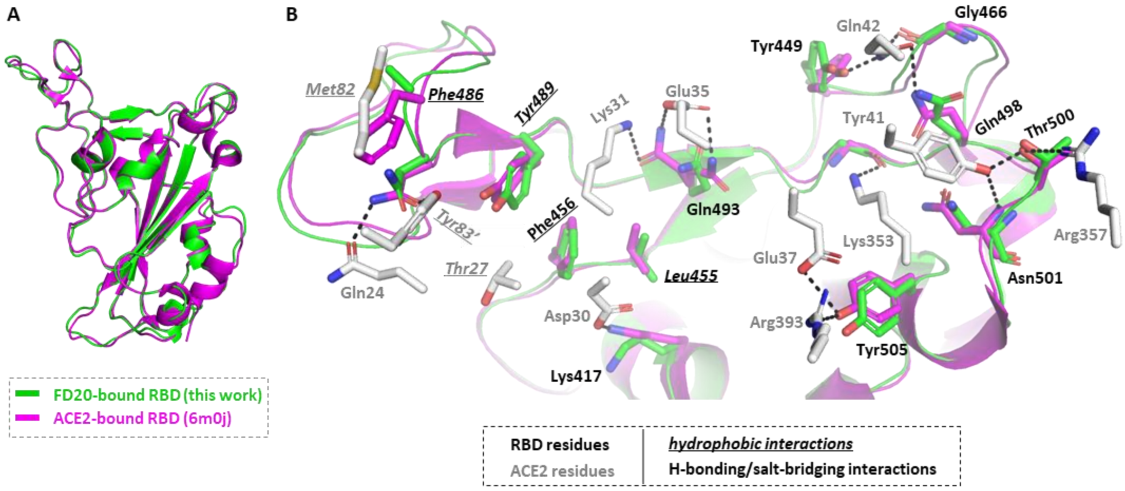

Appendix Fig S2. Comparison of RBD conformation between the FD20-bound form and the ACE2-bound form.

(**A**) The overall similarity of the RBD structure between the FD20-bound form (green) and the ACE2-bound form (magenta). The Cα root mean square deviation of the two structures is 0.669 Å.

(**B**) The slight differences of the RBD structures between the FD20-bound form (green) and the ACE2-bound form (magenta). Residues from ACE2 at the receptor-binding motif (RBM) are shown as grey sticks and are also labeled with grey texts. RBM residues are labeled as black texts. Residues involved in the binding by hydrophobic interactions are labeled as underline and italic texts.

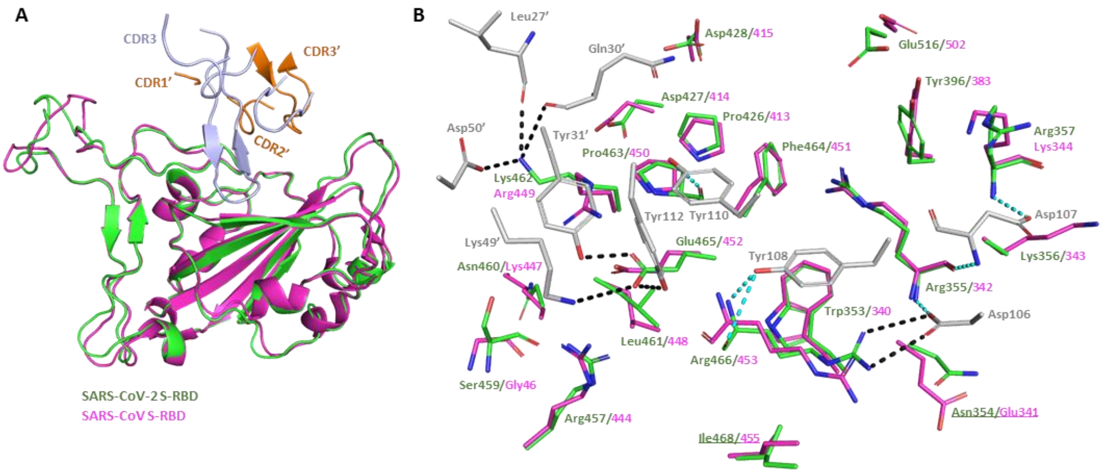

C

```
                                   339       349       359       369       379       389       399       409
SARS-CoV-2          330  PNITNLCPFG EVFNATRFAS VYAWNRKRIS NCVADYSVLY NSASFSTFKC YGVSPTKLND LCFTNVYADS FVIRGDEVRQ
RaTG13  (89.5%)     330  .......... ......T... .......... .......... ..T....... .......... .......... ...T......
SARS-CoV  (74.0%)   317  .......... ......K.P. ....E..K.. .......... ..TF...... ....A..... ...S...... ..VK..D...
MERS-CoV  (21.4%)   379  --EGVE.D.S PLLSG-TPPQ ..NFK.LVFT ..NYNLTK.L SLF.VND.T. SQI..AAIAS N.YSSLIL.Y .SYPLSMKSD
hCoV-OC43  (17.9%)  330  ..LP-N.NIE AWL.DKSVP. PLN.E..TF. ..NFNM.S.M SFIQADS.T. NNIDAA.IYG M..SSITI.K .A.PNGRKVD
hCoV-HKU1  (15.8%)  322  .DLP-D.DID KWL.NFNVP. PLN.E..IF. ..NFNL.T.L RLVHTDS.S. NNFDES.IYG S..KSIVL.K .A.PNSRRSD

                                   419       429       439       446                                  448      457
SARS-CoV-2          410  IAPGQTGKIA DYNYKLPDDF TGCVIAWNSN NLDS---KVG ---------- ---------- --------GN YNYLYRLFR-
RaTG13  (89.5%)     410  .......... .....L.... .........K HI.A---.E. ---------- ---------- --------.. F...Y....-
SARS-CoV  (74.0%)   397  .......V.. .....L.... M...L...TR .I.A---TST ---------- ---------- --------.. ...KY.YL.-
MERS-CoV  (21.4%)   456  LSVSSA.P.S QF...QSFSN PT.L.LATVP HNLTTIT.PL NYNLKYSYIN KCSRLLSDDR TEVPQ----- ----------
hCoV-OC43  (17.9%)  409  LQL.NL.YLQ SF..RIDTTA .S.QLYY.LP AANV---S.S ---PSTWNKR FG-FIEDSVF KPRPAGVLT. HDVVYAQHCF
hCoV-HKU1  (15.8%)  401  LQL.SS.FLQ SS...IDTTS SS.QLYYSLP AINV---TIN RFNPSSWNRR YGFNN----- ----FNLSSH SVVYS.YCFS

                                                                                                  458      466
SARS-CoV-2          458  ---------- ---------- ---------- ---------- ---------- ---------- ---------K SNLKPFER--
RaTG13  (89.5%)     458  ---------- ---------- ---------- ---------- ---------- ---------- ---------. A.......--
SARS-CoV  (74.0%)   445  ---------- ---------- ---------- ---------- ---------- ---------- ---------H GK.R....--
MERS-CoV  (21.4%)   516  ---------- ---------- ---------- ---------- ---------- ---------- -------Q-- -LVNANQYSP
hCoV-OC43  (17.9%)  485  KAPKNFCPCK LNGSCVGSGP GKNNGIGTCP AGTNYLTCDN LCTPDPITFT GTYKCP---- -------QT. .LVGIG.H--
hCoV-HKU1  (15.8%)  469  VNNTFCPCAK P-SFASSCKS HKPPSASCPI GTNYRSCEST TVLDHTDWCR CSCLPDPITA YDPRSCSQK. .LVGVG.H--

                                   476       483       491       501       511      520
SARS-CoV-2          467  DISTEIYQAG STPC---NGV EGFNC--YFP LQSYGFQPTN GVGYQPYRVV VLSFELLHA- -PATVCGPKK
RaTG13  (89.5%)     467  .......... .K..---..Q TGL..--.Y. .YR...Y..D ...H...... .......N.- -.....G...
SARS-CoV  (74.0%)   454  ...NVPFSPD GK..---T-P PAL..--.W. .ND...YT.T .I........ .......N.- -.....G..L
MERS-CoV  (21.4%)   526  CV.I-VPSTV WEDGDYYRKQ LSPLEGGGWL VASGSTVAMT EQLQMG---F GITVQYGTDT NS--V.-..L
hCoV-OC43  (17.9%)  552  CSGLAVKSDY CGGNS----- -CTCRPQAF- .GWSADS--- -CLQGDKCNI FAN.I.HDVN SGL.-.STDL
hCoV-HKU1  (15.8%)  546  CAGFGVDEEK CGVLDGSYNV SCLCSTDAF- .GWSYDT--- -CVSNNRCNI FSN.I.NGIN SGT.-.SDLL
```

FD20 epitope
Highly conservative
Modestly conservative
Previously predicted ideal epitope

Appendix Fig S3. Structural comparison of RBD-equivalent regions in other coronaviruses.

(**A, B**) Aligning scFD20-RBD to the structure of SARS-CoV RBD in complex with a neutralizing antibody M396. The Cα root mean square deviation of the two structures is 0.669 Å. The similarity in the overall structure (**A**) and at the epitope region (**B**) is shown.

(**C**) The FD20 epitope (red) is conserved between SARS-CoV-2, RaTG13, and SARS-CoV. Dots indicate identical residues and dashes indicate gaps. Brackets indicate sequence identity between the aligned RBD and SARS-CoV-2 RBD. NCBI accession codes for the sequences are as follows. RaTG13, QHR63300.2; SARS-CoV, NC_004718.3; MERS-CoV, NC_019843.3; hCoV-OC43, NC_006213.1; hCoV-HKU1, NC_006577.2.

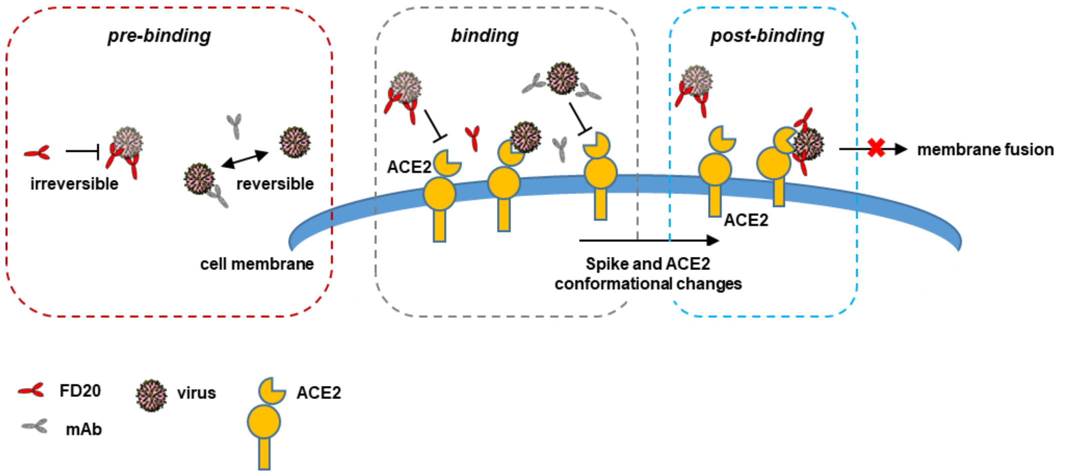

Appendix Fig S4. Schematic mechanism diagram of antibodies that act on the *pre-binding*, *binding*, and *post-binding* steps.

Full wwPDB X-ray Structure Validation Report (i)

Sep 6, 2021 – 02:07 PM JST

PDB ID	:	7CYV
Title	:	Crystal structure of FD20, a neutralizing single-chain variable fragment (scFv) in complex with SARS-CoV-2 Spike receptor-binding domain (RBD)
Authors	:	Li, Y.; Li, T.; Lai, Y.; Cai, H.; Yao, H.; Li, D.
Deposited on	:	2020-09-04
Resolution	:	3.13 Å(reported)

This is a Full wwPDB X-ray Structure Validation Report for a publicly released PDB entry.

We welcome your comments at *validation@mail.wwpdb.org*
A user guide is available at
https://www.wwpdb.org/validation/2017/XrayValidationReportHelp
with specific help available everywhere you see the (i) symbol.

The following versions of software and data (see references (i)) were used in the production of this report:

MolProbity	:	4.02b-467
Mogul	:	1.8.5 (274361), CSD as541be (2020)
Xtriage (Phenix)	:	1.13
EDS	:	2.23.1
Percentile statistics	:	20191225.v01 (using entries in the PDB archive December 25th 2019)
Refmac	:	5.8.0158
CCP4	:	7.0.044 (Gargrove)
Ideal geometry (proteins)	:	Engh & Huber (2001)
Ideal geometry (DNA, RNA)	:	Parkinson et al. (1996)
Validation Pipeline (wwPDB-VP)	:	2.23.1

1 Overall quality at a glance (i)

The following experimental techniques were used to determine the structure:
X-RAY DIFFRACTION

The reported resolution of this entry is 3.13 Å.

Percentile scores (ranging between 0-100) for global validation metrics of the entry are shown in the following graphic. The table shows the number of entries on which the scores are based.

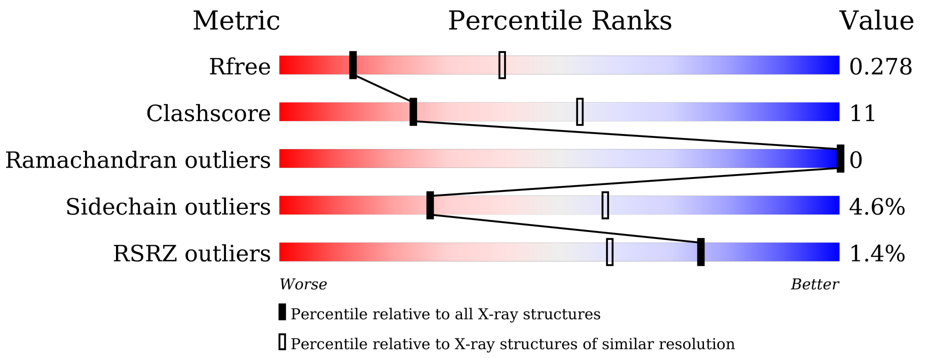

Metric	Whole archive (#Entries)	Similar resolution (#Entries, resolution range(Å))
R_{free}	130704	1626 (3.18-3.10)
Clashscore	141614	1735 (3.18-3.10)
Ramachandran outliers	138981	1677 (3.18-3.10)
Sidechain outliers	138945	1677 (3.18-3.10)
RSRZ outliers	127900	1588 (3.18-3.10)

The table below summarises the geometric issues observed across the polymeric chains and their fit to the electron density. The red, orange, yellow and green segments of the lower bar indicate the fraction of residues that contain outliers for >=3, 2, 1 and 0 types of geometric quality criteria respectively. A grey segment represents the fraction of residues that are not modelled. The numeric value for each fraction is indicated below the corresponding segment, with a dot representing fractions <=5% The upper red bar (where present) indicates the fraction of residues that have poor fit to the electron density. The numeric value is given above the bar.

Mol	Chain	Length	Quality of chain
1	H	281	2% / 62% 22% • 16%
2	B	213	69% 20% • 9%
3	A	5	60% 40%

2 Entry composition

There are 3 unique types of molecules in this entry. The entry contains 3225 atoms, of which 0 are hydrogens and 0 are deuteriums.

In the tables below, the ZeroOcc column contains the number of atoms modelled with zero occupancy, the AltConf column contains the number of residues with at least one atom in alternate conformation and the Trace column contains the number of residues modelled with at most 2 atoms.

- Molecule 1 is a protein called The heavy chain variable region of the scFv FD20,The light chain variable region of the scFv FD20.

Mol	Chain	Residues	Atoms					ZeroOcc	AltConf	Trace
			Total	C	N	O	S			
1	H	236	1700	1085	275	332	8	0	0	0

- Molecule 2 is a protein called Spike protein S1.

Mol	Chain	Residues	Atoms						ZeroOcc	AltConf	Trace
			Total	C	N	O	P	S			
2	B	193	1466	937	248	272	1	8	0	0	0

There are 11 discrepancies between the modelled and reference sequences:

Chain	Residue	Modelled	Actual	Comment	Reference
B	327	ALA	-	expression tag	UNP P0DTC2
B	328	GLY	-	expression tag	UNP P0DTC2
B	329	SER	-	expression tag	UNP P0DTC2
B	532	GLY	-	expression tag	UNP P0DTC2
B	533	THR	-	expression tag	UNP P0DTC2
B	534	LEU	-	expression tag	UNP P0DTC2
B	535	GLU	-	expression tag	UNP P0DTC2
B	536	VAL	-	expression tag	UNP P0DTC2
B	537	LEU	-	expression tag	UNP P0DTC2
B	538	PHE	-	expression tag	UNP P0DTC2
B	539	GLN	-	expression tag	UNP P0DTC2

- Molecule 3 is an oligosaccharide called beta-D-mannopyranose-(1-4)-2-acetamido-2-deoxy-beta-D-glucopyranose-(1-4)-[alpha-L-fucopyranose-(1-3)][alpha-L-fucopyranose-(1-6)]2-acetamido-2-deoxy-beta-D-glucopyranose.

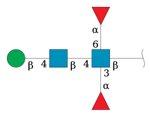

Mol	Chain	Residues	Atoms				ZeroOcc	AltConf	Trace
			Total	C	N	O			
3	A	5	59	34	2	23	0	0	0

3 Residue-property plots ⓘ

These plots are drawn for all protein, RNA, DNA and oligosaccharide chains in the entry. The first graphic for a chain summarises the proportions of the various outlier classes displayed in the second graphic. The second graphic shows the sequence view annotated by issues in geometry and electron density. Residues are color-coded according to the number of geometric quality criteria for which they contain at least one outlier: green = 0, yellow = 1, orange = 2 and red = 3 or more. A red dot above a residue indicates a poor fit to the electron density (RSRZ > 2). Stretches of 2 or more consecutive residues without any outlier are shown as a green connector. Residues present in the sample, but not in the model, are shown in grey.

- Molecule 1: The heavy chain variable region of the scFv FD20,The light chain variable region of the scFv FD20

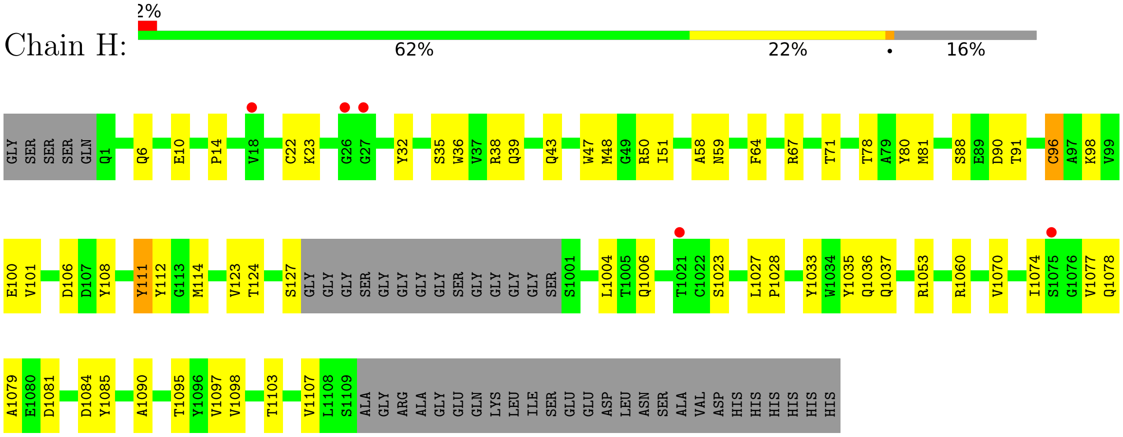

- Molecule 2: Spike protein S1

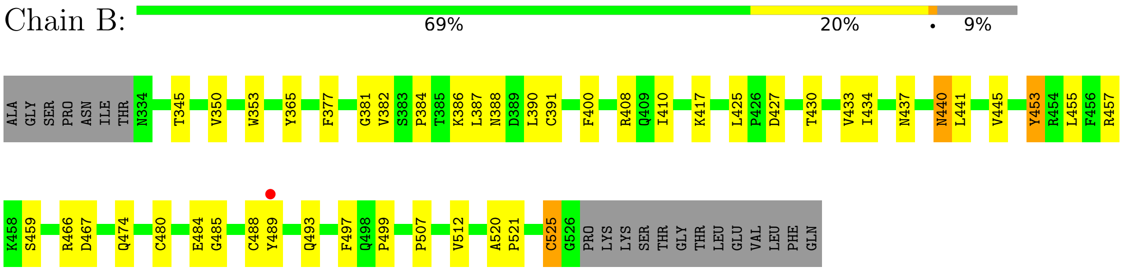

- Molecule 3: beta-D-mannopyranose-(1-4)-2-acetamido-2-deoxy-beta-D-glucopyranose-(1-4)-[alpha-L-fucopyranose-(1-3)][alpha-L-fucopyranose-(1-6)]2-acetamido-2-deoxy-beta-D-glucopyranose

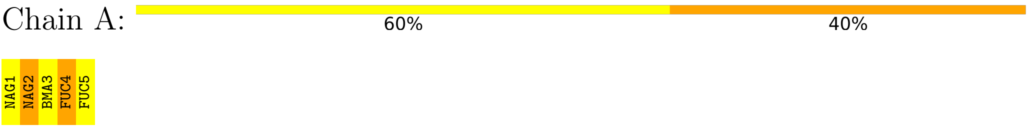

4 Data and refinement statistics (i)

Property	Value	Source
Space group	C 1 2 1	Depositor
Cell constants<br>a, b, c, α, β, γ	206.96Å 57.92Å 47.21Å<br>90.00° 100.43° 90.00°	Depositor
Resolution (Å)	46.43 – 3.13<br>46.43 – 3.13	Depositor<br>EDS
% Data completeness<br>(in resolution range)	98.9 (46.43-3.13)<br>99.2 (46.43-3.13)	Depositor<br>EDS
R_{merge}	0.27	Depositor
R_{sym}	(Not available)	Depositor
$< I/\sigma(I) >$ [1]	1.28 (at 3.12Å)	Xtriage
Refinement program	PHENIX 1.17.1_3660	Depositor
R, R_{free}	0.252 , 0.276<br>0.253 , 0.278	Depositor<br>DCC
R_{free} test set	463 reflections (4.73%)	wwPDB-VP
Wilson B-factor (Å^2)	68.8	Xtriage
Anisotropy	0.409	Xtriage
Bulk solvent k_{sol}(e/Å^3), B_{sol}(Å^2)	0.30 , 35.9	EDS
L-test for twinning[2]	$< \|L\| >$ = 0.48, $< L^2 >$ = 0.31	Xtriage
Estimated twinning fraction	0.030 for -h-2*l,-k,l	Xtriage
F_o,F_c correlation	0.90	EDS
Total number of atoms	3225	wwPDB-VP
Average B, all atoms (Å^2)	61.0	wwPDB-VP

Xtriage's analysis on translational NCS is as follows: *The largest off-origin peak in the Patterson function is 7.08% of the height of the origin peak. No significant pseudotranslation is detected.*

[1] Intensities estimated from amplitudes.

[2] Theoretical values of $< |L| >$, $< L^2 >$ for acentric reflections are 0.5, 0.333 respectively for untwinned datasets, and 0.375, 0.2 for perfectly twinned datasets.

5 Model quality (i)

5.1 Standard geometry (i)

Bond lengths and bond angles in the following residue types are not validated in this section: BMA, TPO, FUC, NAG

The Z score for a bond length (or angle) is the number of standard deviations the observed value is removed from the expected value. A bond length (or angle) with $|Z| > 5$ is considered an outlier worth inspection. RMSZ is the root-mean-square of all Z scores of the bond lengths (or angles).

Mol	Chain	Bond lengths		Bond angles	
		RMSZ	$\#\|Z\| >5$	RMSZ	$\#\|Z\| >5$
1	H	0.28	0/1740	0.50	0/2383
2	B	0.29	0/1495	0.52	0/2040
All	All	0.29	0/3235	0.51	0/4423

There are no bond length outliers.

There are no bond angle outliers.

There are no chirality outliers.

There are no planarity outliers.

5.2 Too-close contacts (i)

In the following table, the Non-H and H(model) columns list the number of non-hydrogen atoms and hydrogen atoms in the chain respectively. The H(added) column lists the number of hydrogen atoms added and optimized by MolProbity. The Clashes column lists the number of clashes within the asymmetric unit, whereas Symm-Clashes lists symmetry-related clashes.

Mol	Chain	Non-H	H(model)	H(added)	Clashes	Symm-Clashes
1	H	1700	0	1560	42	0
2	B	1466	0	1315	25	0
3	A	59	0	52	1	0
All	All	3225	0	2927	68	0

The all-atom clashscore is defined as the number of clashes found per 1000 atoms (including hydrogen atoms). The all-atom clashscore for this structure is 11.

All (68) close contacts within the same asymmetric unit are listed below, sorted by their clash magnitude.

Atom-1	Atom-2	Interatomic distance (Å)	Clash overlap (Å)
3:A:2:NAG:H61	3:A:4:FUC:H63	1.62	0.81
2:B:391:CYS:HA	2:B:525:CYS:HB3	1.69	0.73
1:H:38:ARG:NH1	1:H:90:ASP:OD1	2.22	0.71
1:H:39:GLN:OE1	1:H:1037:GLN:NE2	2.22	0.71
1:H:47:TRP:CD2	1:H:1097:VAL:HG22	2.26	0.71
1:H:1090:ALA:HA	1:H:1097:VAL:HA	1.75	0.67
2:B:365:TYR:HD2	2:B:388:ASN:HA	1.60	0.67
1:H:48:MET:HG2	1:H:64:PHE:HE2	1.60	0.64
1:H:51:ILE:HG13	1:H:58:ALA:HB2	1.82	0.62
2:B:417:LYS:HD2	2:B:455:LEU:HD12	1.82	0.62
1:H:1060:ARG:NH1	1:H:1081:ASP:OD2	2.33	0.61
1:H:39:GLN:NE2	1:H:43:GLN:O	2.31	0.61
1:H:1074:ILE:HB	1:H:1077:VAL:HG12	1.84	0.60
1:H:1079:ALA:HA	1:H:1107:VAL:HG11	1.84	0.59
2:B:484:GLU:OE2	2:B:484:GLU:N	2.33	0.59
2:B:433:VAL:HG22	2:B:512:VAL:HG13	1.86	0.57
1:H:47:TRP:HZ2	1:H:50:ARG:HG2	1.69	0.57
1:H:67:ARG:NH2	1:H:90:ASP:OD2	2.38	0.56
1:H:35:SER:OG	1:H:47:TRP:NE1	2.37	0.56
1:H:6:GLN:HG2	1:H:22:CYS:HB3	1.89	0.54
1:H:59:ASN:ND2	1:H:1095:THR:O	2.40	0.54
1:H:100:GLU:O	1:H:112:TYR:HB2	2.06	0.54
2:B:365:TYR:CD2	2:B:388:ASN:HA	2.41	0.54
1:H:1036:GLN:HB2	1:H:1085:TYR:CE1	2.43	0.53
1:H:14:PRO:HG2	1:H:127:SER:HB2	1.90	0.53
1:H:50:ARG:HD2	1:H:111:TYR:CZ	2.44	0.52
2:B:386:LYS:O	2:B:390:LEU:HD23	2.10	0.52
2:B:457:ARG:HD3	2:B:459:SER:O	2.10	0.52
1:H:1006:GLN:NE2	1:H:1085:TYR:O	2.42	0.52
2:B:497:PHE:CE2	2:B:507:PRO:HB3	2.45	0.52
1:H:6:GLN:HA	1:H:22:CYS:HA	1.93	0.51
1:H:106:ASP:O	1:H:108:TYR:N	2.43	0.51
1:H:50:ARG:HD2	1:H:111:TYR:CE1	2.46	0.51
2:B:520:ALA:HB1	2:B:521:PRO:HD2	1.92	0.50
2:B:485:GLY:N	2:B:488:CYS:O	2.42	0.50
1:H:47:TRP:CG	1:H:1097:VAL:HG22	2.46	0.50
1:H:112:TYR:HA	1:H:1033:TYR:OH	2.12	0.50
2:B:391:CYS:CA	2:B:525:CYS:HB3	2.34	0.50
1:H:23:LYS:HG3	1:H:78:THR:HG22	1.94	0.49
1:H:1027:LEU:HD23	1:H:1070:VAL:HG23	1.93	0.49
2:B:474:GLN:HA	2:B:480:CYS:SG	2.52	0.49
2:B:381:GLY:HA3	2:B:430:THR:HA	1.94	0.48

Continued on next page...

Continued from previous page...

Atom-1	Atom-2	Interatomic distance (Å)	Clash overlap (Å)
1:H:71:THR:HG23	1:H:80:TYR:HB2	1.95	0.48
1:H:10:GLU:HB2	1:H:123:VAL:HG22	1.96	0.47
2:B:453:TYR:CE1	2:B:493:GLN:HB3	2.49	0.47
2:B:457:ARG:HH11	2:B:467:ASP:HB2	1.79	0.47
1:H:35:SER:HG	1:H:47:TRP:HE1	1.59	0.47
2:B:350:VAL:HA	2:B:400:PHE:HB2	1.97	0.46
1:H:1027:LEU:N	1:H:1028:PRO:HD2	2.31	0.46
1:H:36:TRP:CE2	1:H:81:MET:HB2	2.52	0.45
1:H:101:VAL:HG23	1:H:111:TYR:HA	1.98	0.45
1:H:1006:GLN:HG2	1:H:1103:THR:OG1	2.17	0.45
2:B:377:PHE:CD2	2:B:434:ILE:HG12	2.52	0.45
1:H:98:LYS:HB3	1:H:98:LYS:HE3	1.76	0.44
2:B:440:ASN:OD1	2:B:441:LEU:HG	2.18	0.44
2:B:353:TRP:NE1	2:B:466:ARG:HB2	2.33	0.43
2:B:410:ILE:O	2:B:425:LEU:HD13	2.18	0.43
1:H:1004:LEU:HG	1:H:1098:VAL:HG13	2.00	0.43
1:H:32:TYR:CD1	1:H:98:LYS:HD3	2.55	0.42
1:H:36:TRP:CH2	1:H:96:CYS:HB2	2.55	0.42
1:H:91:THR:HG23	1:H:124:THR:HA	2.02	0.41
2:B:384:PRO:HA	2:B:387:LEU:HB2	2.01	0.41
2:B:382:VAL:HG21	2:B:390:LEU:HG	2.01	0.41
1:H:114:MET:O	1:H:1035:TYR:HE1	2.04	0.41
2:B:445:VAL:HG22	2:B:499:PRO:HG2	2.03	0.41
2:B:497:PHE:CZ	2:B:507:PRO:HB3	2.54	0.41
1:H:35:SER:HA	1:H:50:ARG:HA	2.03	0.40
1:H:36:TRP:HB3	1:H:48:MET:HE2	2.02	0.40

There are no symmetry-related clashes.

5.3 Torsion angles (i)

5.3.1 Protein backbone (i)

In the following table, the Percentiles column shows the percent Ramachandran outliers of the chain as a percentile score with respect to all X-ray entries followed by that with respect to entries of similar resolution.

The Analysed column shows the number of residues for which the backbone conformation was analysed, and the total number of residues.

Mol	Chain	Analysed	Favoured	Allowed	Outliers	Percentiles	
1	H	232/281 (83%)	224 (97%)	8 (3%)	0	100	100
2	B	190/213 (89%)	181 (95%)	9 (5%)	0	100	100
All	All	422/494 (85%)	405 (96%)	17 (4%)	0	100	100

There are no Ramachandran outliers to report.

5.3.2 Protein sidechains (i)

In the following table, the Percentiles column shows the percent sidechain outliers of the chain as a percentile score with respect to all X-ray entries followed by that with respect to entries of similar resolution.

The Analysed column shows the number of residues for which the sidechain conformation was analysed, and the total number of residues.

Mol	Chain	Analysed	Rotameric	Outliers	Percentiles	
1	H	164/222 (74%)	157 (96%)	7 (4%)	29	60
2	B	143/182 (79%)	136 (95%)	7 (5%)	25	56
All	All	307/404 (76%)	293 (95%)	14 (5%)	27	58

All (14) residues with a non-rotameric sidechain are listed below:

Mol	Chain	Res	Type
1	H	88	SER
1	H	96	CYS
1	H	111	TYR
1	H	1023	SER
1	H	1053	ARG
1	H	1078	GLN
1	H	1084	ASP
2	B	408	ARG
2	B	427	ASP
2	B	437	ASN
2	B	440	ASN
2	B	453	TYR
2	B	489	TYR
2	B	525	CYS

Sometimes sidechains can be flipped to improve hydrogen bonding and reduce clashes. All (5) such sidechains are listed below:

Mol	Chain	Res	Type
1	H	65	GLN
1	H	1036	GLN
2	B	394	ASN
2	B	409	GLN
2	B	437	ASN

5.3.3 RNA ⓘ

There are no RNA molecules in this entry.

5.4 Non-standard residues in protein, DNA, RNA chains ⓘ

1 non-standard protein/DNA/RNA residue is modelled in this entry.

In the following table, the Counts columns list the number of bonds (or angles) for which Mogul statistics could be retrieved, the number of bonds (or angles) that are observed in the model and the number of bonds (or angles) that are defined in the Chemical Component Dictionary. The Link column lists molecule types, if any, to which the group is linked. The Z score for a bond length (or angle) is the number of standard deviations the observed value is removed from the expected value. A bond length (or angle) with $|Z| > 2$ is considered an outlier worth inspection. RMSZ is the root-mean-square of all Z scores of the bond lengths (or angles).

Mol	Type	Chain	Res	Link	Bond lengths			Bond angles		
					Counts	RMSZ	$\#\|Z\| > 2$	Counts	RMSZ	$\#\|Z\| > 2$
2	TPO	B	345	2	8,10,11	1.18	0	10,14,16	1.13	1 (10%)

In the following table, the Chirals column lists the number of chiral outliers, the number of chiral centers analysed, the number of these observed in the model and the number defined in the Chemical Component Dictionary. Similar counts are reported in the Torsion and Rings columns. '-' means no outliers of that kind were identified.

Mol	Type	Chain	Res	Link	Chirals	Torsions	Rings
2	TPO	B	345	2	-	4/9/11/13	-

There are no bond length outliers.

All (1) bond angle outliers are listed below:

Mol	Chain	Res	Type	Atoms	Z	Observed(^{o})	Ideal(^{o})
2	B	345	TPO	O3P-P-OG1	2.36	116.57	105.99

There are no chirality outliers.

All (4) torsion outliers are listed below:

Mol	Chain	Res	Type	Atoms
2	B	345	TPO	O-C-CA-CB
2	B	345	TPO	CG2-CB-OG1-P
2	B	345	TPO	CB-OG1-P-O1P
2	B	345	TPO	CB-OG1-P-O3P

There are no ring outliers.

No monomer is involved in short contacts.

5.5 Carbohydrates (i)

5 monosaccharides are modelled in this entry.

In the following table, the Counts columns list the number of bonds (or angles) for which Mogul statistics could be retrieved, the number of bonds (or angles) that are observed in the model and the number of bonds (or angles) that are defined in the Chemical Component Dictionary. The Link column lists molecule types, if any, to which the group is linked. The Z score for a bond length (or angle) is the number of standard deviations the observed value is removed from the expected value. A bond length (or angle) with $|Z| > 2$ is considered an outlier worth inspection. RMSZ is the root-mean-square of all Z scores of the bond lengths (or angles).

Mol	Type	Chain	Res	Link	Bond lengths			Bond angles		
					Counts	RMSZ	$\#\|Z\| > 2$	Counts	RMSZ	$\#\|Z\| > 2$
3	NAG	A	1	3,2	14,14,15	0.25	0	17,19,21	1.80	4 (23%)
3	NAG	A	2	3	14,14,15	0.64	1 (7%)	17,19,21	0.86	0
3	BMA	A	3	3	11,11,12	0.73	1 (9%)	15,15,17	1.21	1 (6%)
3	FUC	A	4	3	10,10,11	1.38	1 (10%)	14,14,16	1.99	4 (28%)
3	FUC	A	5	3	10,10,11	0.69	0	14,14,16	0.99	1 (7%)

In the following table, the Chirals column lists the number of chiral outliers, the number of chiral centers analysed, the number of these observed in the model and the number defined in the Chemical Component Dictionary. Similar counts are reported in the Torsion and Rings columns. '-' means no outliers of that kind were identified.

Mol	Type	Chain	Res	Link	Chirals	Torsions	Rings
3	NAG	A	1	3,2	-	1/6/23/26	0/1/1/1
3	NAG	A	2	3	-	0/6/23/26	0/1/1/1
3	BMA	A	3	3	-	2/2/19/22	0/1/1/1
3	FUC	A	4	3	-	-	0/1/1/1
3	FUC	A	5	3	-	-	0/1/1/1

All (3) bond length outliers are listed below:

Mol	Chain	Res	Type	Atoms	Z	Observed(Å)	Ideal(Å)
3	A	4	FUC	C1-C2	3.55	1.60	1.52
3	A	3	BMA	C1-C2	2.31	1.57	1.52
3	A	2	NAG	C1-C2	2.08	1.55	1.52

All (10) bond angle outliers are listed below:

Mol	Chain	Res	Type	Atoms	Z	Observed(^{o})	Ideal(^{o})
3	A	4	FUC	O5-C1-C2	4.01	116.96	110.77
3	A	1	NAG	O3-C3-C4	3.92	119.41	110.35
3	A	1	NAG	C1-O5-C5	3.89	117.46	112.19
3	A	4	FUC	C1-C2-C3	3.35	113.78	109.67
3	A	4	FUC	C1-O5-C5	3.20	120.04	112.78
3	A	3	BMA	C1-O5-C5	2.74	115.91	112.19
3	A	4	FUC	O5-C5-C4	2.54	114.08	109.52
3	A	1	NAG	C4-C3-C2	-2.33	107.60	111.02
3	A	1	NAG	C2-N2-C7	2.15	125.97	122.90
3	A	5	FUC	C1-O5-C5	2.06	117.45	112.78

There are no chirality outliers.

All (3) torsion outliers are listed below:

Mol	Chain	Res	Type	Atoms
3	A	3	BMA	O5-C5-C6-O6
3	A	3	BMA	C4-C5-C6-O6
3	A	1	NAG	C3-C2-N2-C7

There are no ring outliers.

2 monomers are involved in 1 short contact:

Mol	Chain	Res	Type	Clashes	Symm-Clashes
3	A	2	NAG	1	0
3	A	4	FUC	1	0

The following is a two-dimensional graphical depiction of Mogul quality analysis of bond lengths, bond angles, torsion angles, and ring geometry for oligosaccharide.

Oligosaccharide Chain A

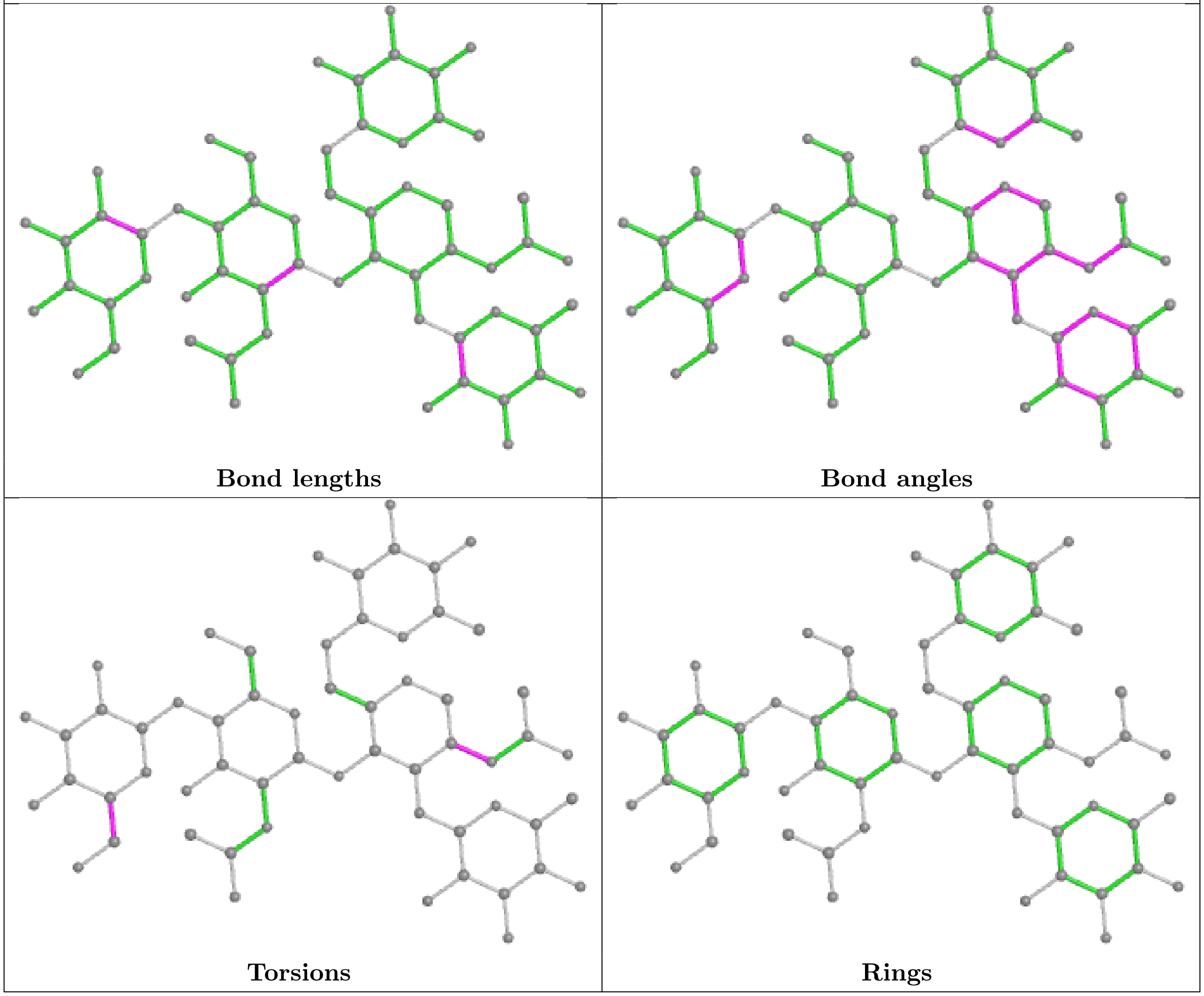

5.6 Ligand geometry (i)

There are no ligands in this entry.

5.7 Other polymers (i)

There are no such residues in this entry.

5.8 Polymer linkage issues (i)

There are no chain breaks in this entry.

6 Fit of model and data (i)

6.1 Protein, DNA and RNA chains (i)

In the following table, the column labelled '#RSRZ> 2' contains the number (and percentage) of RSRZ outliers, followed by percent RSRZ outliers for the chain as percentile scores relative to all X-ray entries and entries of similar resolution. The OWAB column contains the minimum, median, 95th percentile and maximum values of the occupancy-weighted average B-factor per residue. The column labelled 'Q< 0.9' lists the number of (and percentage) of residues with an average occupancy less than 0.9.

Mol	Chain	Analysed	<RSRZ>	#RSRZ>2			OWAB(Å^2)	Q<0.9
1	H	236/281 (83%)	0.04	5 (2%)	63	44	45, 61, 80, 96	1 (0%)
2	B	192/213 (90%)	-0.03	1 (0%)	91	83	40, 56, 101, 121	0
All	All	428/494 (86%)	0.01	6 (1%)	75	59	40, 60, 94, 121	1 (0%)

All (6) RSRZ outliers are listed below:

Mol	Chain	Res	Type	RSRZ
1	H	26	GLY	2.5
2	B	489	TYR	2.3
1	H	27	GLY	2.3
1	H	18	VAL	2.3
1	H	1021	THR	2.3
1	H	1075	SER	2.1

6.2 Non-standard residues in protein, DNA, RNA chains (i)

In the following table, the Atoms column lists the number of modelled atoms in the group and the number defined in the chemical component dictionary. The B-factors column lists the minimum, median, 95th percentile and maximum values of B factors of atoms in the group. The column labelled 'Q< 0.9' lists the number of atoms with occupancy less than 0.9.

Mol	Type	Chain	Res	Atoms	RSCC	RSR	B-factors(Å^2)	Q<0.9
2	TPO	B	345	11/12	0.83	0.22	57,57,57,57	0

6.3 Carbohydrates (i)

In the following table, the Atoms column lists the number of modelled atoms in the group and the number defined in the chemical component dictionary. The B-factors column lists the minimum, median, 95th percentile and maximum values of B factors of atoms in the group. The column labelled 'Q< 0.9' lists the number of atoms with occupancy less than 0.9.

Mol	Type	Chain	Res	Atoms	RSCC	RSR	B-factors(Å^2)	Q<0.9
3	BMA	A	3	11/12	0.60	0.33	98,98,98,98	0
3	NAG	A	2	14/15	0.82	0.21	85,85,85,85	0
3	FUC	A	4	10/11	0.82	0.29	86,86,86,86	0
3	NAG	A	1	14/15	0.87	0.22	76,76,76,76	0
3	FUC	A	5	10/11	0.87	0.22	77,77,77,77	0

The following is a graphical depiction of the model fit to experimental electron density for oligosaccharide. Each fit is shown from different orientation to approximate a three-dimensional view.

Electron density around Chain A:

2mF$_o$-DF$_c$ (at 0.7 rmsd) in gray
mF$_o$-DF$_c$ (at 3 rmsd) in purple (negative)
and green (positive)

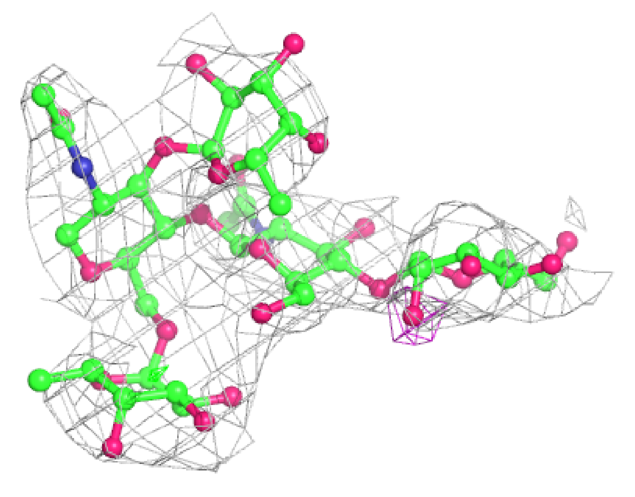

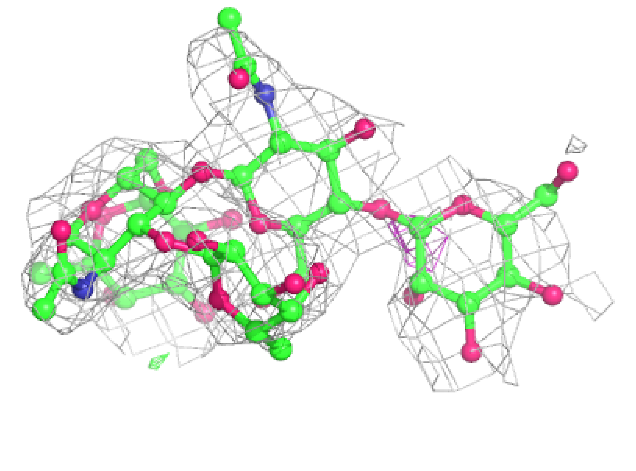

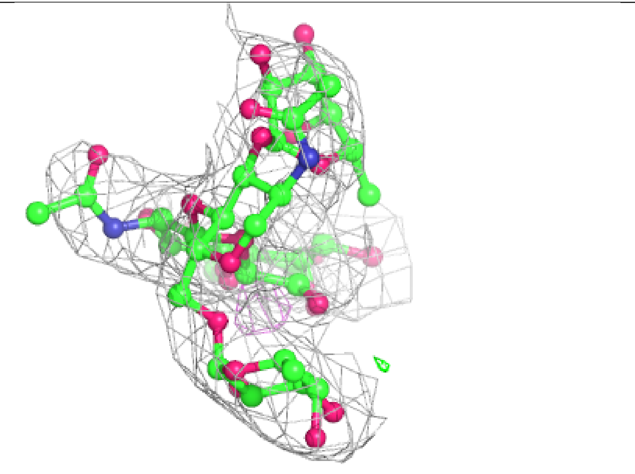

6.4 Ligands ⓘ

There are no ligands in this entry.

6.5 Other polymers ⓘ

There are no such residues in this entry.
