# Supplementary material for: Uncovering a conserved vulnerability site in SARS‐CoV‐2 by a human antibody
Source: EMBO Mol Med. 2021 Nov 17;13(12):e14544. doi: 10.15252/emmm.202114544 (PMC8646660; doi:10.15252/emmm.202114544)
Supplement: Supplementary file 2 — Expanded View Figures PDF [file EMMM-13-e14544-s001.pdf]

Expanded View Figures

A

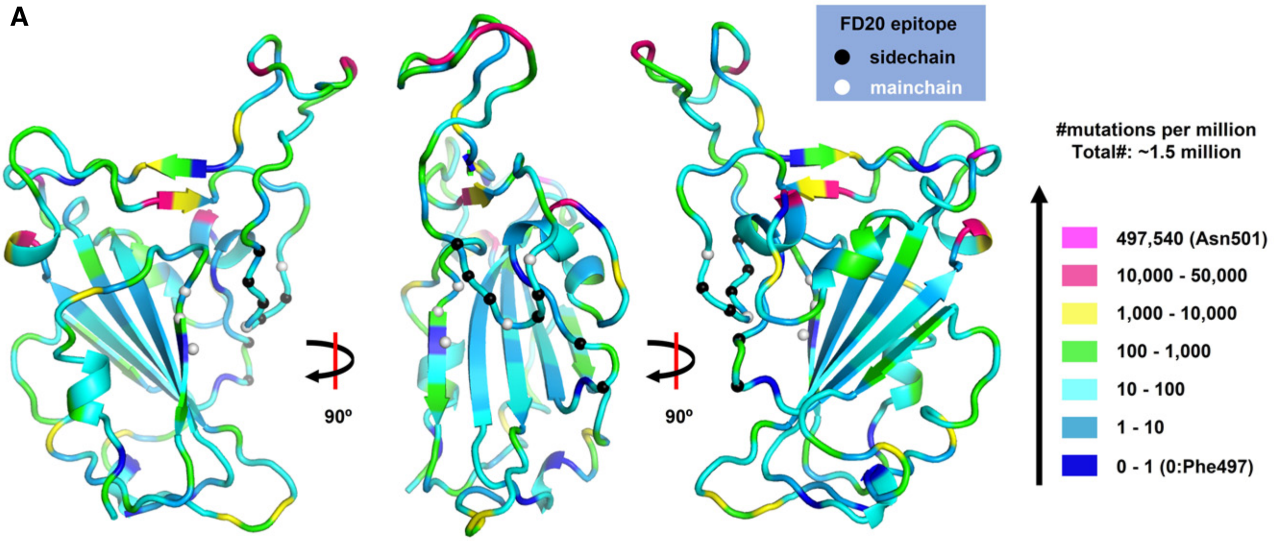

B

Position	Wild-type	Mutations [counts][a]	#mutations per million	Interaction type
353	Trp	Arg [4]; Cys [1]; Phe [1]	4.1	main chain
354	Asn	Lys [252]; Asp [200]; His [66]; Ser [45]; Ile [8]; Thr [3]	391.7	main chain
355	Arg	Thr [1]	0.7	main chain
426	Pro	Ser [21]; Thr [2]; Gln [1]	16.3	side chain
428	Asp	Gly [22]; Tyr [8]; Glu [7]; His [2]; Val [2]; Ala [1]; Ile [1]	29.3	side chain
461	Leu	Ile [13]; Phe [2]	10.2	main chain
462	Lys	Arg [8]; Thr [8]; Glu [6]; Gln [2], Asn [1]	21.1	side chain
463	Pro	Ser [83]; Leu [5]; His [2]; Thr [1]	62.0	side chain
464	Phe	Leu [11]; Ser [2]; Ile [1]	9.5	main chain
465	Glu	Asp [21]; Gly [8]; Lys [1]; Gln [1]	21.1	side chain
466	Arg	Lys [10]; Ile [4]; Ser [1]	10.2	side chain
467	Asp	Tyr [2]; Gly [1]; Ile [1]; Lys [1]; Val [1]	4.1	In proximity[b]

[a]From a total of ~1.47 million sequences as of June 9, 2021 (www.gisaid.org; cov.lanl.gov). [b]Near the FD20 epitope.

Figure EV1. Uneven distribution of naturally occurring mutants in RBD.

A The distribution of naturally occurring mutants in RBD viewed at different angles. Residues are color-coded according to their mutation frequency using the color scheme on the right. The FD20 epitopes are labeled as Cα spheres which are color-coded based on their interaction type with FD20 (by side chain or by the main chain).

B Details of the mutations of the FD20 epitope residues.

 © 2021 The Authors

Figure EV2. Mutation of the FD20 epitope residues variably affects binding affinity but has little or no effect on neutralization.

A Biolayer interferometry (BLI) curve of binding between ACE2 and RBD variants. The assay was performed with RBD variants immobilized and ACE2 as analyte at 50 nM.

B BLI curve of binding between FD20 and RBD variants as indicated. The assay was performed with RBD (wild-type and mutants) immobilized and FD20 as analyte at 1 and 5 nM except that only 5 nM was used for K462A/E465A and 50 nM was used for K462E/E465K. The curve for 1 nM was used for the wild-type (WT) in the cases of K462E and E465K. In A and B, the same data for wild-type are repeatedly plotted for visualization reasons.

C The infectivity of SARS-CoV-2 pp harboring indicated S mutants. Mean ± SEM are plotted (n = 5 biological replicates).

D Neutralization assay of FD20 against SARS-CoV-2 pp harboring indicated S mutants. Mean ± SD are plotted (n = 3 biological replicates). Brackets indicate IC_{50} values (nM).

E, F The total expression and the processing of S for the indicated SARS-CoV-2 S mutants. Cell lysates (E) or cell culture supernatants (F) were loaded onto the SDS–PAGE for Western blotting. A red triangle marks the position of S and a blue triangle marks the position of the S1 subunit. Molecular weights are labeled on the left. “-”, empty plasmid; WT, wild-type SARS-CoV-2 plasmid. GAPDH (glyceraldehyde 3-phosphate dehydrogenase) serves as a control for equal loading. Uncropped blots are included in the source data. The results of this figure are summarized in Table 2.

Source data are available online for this figure.

© 2021 The Authors

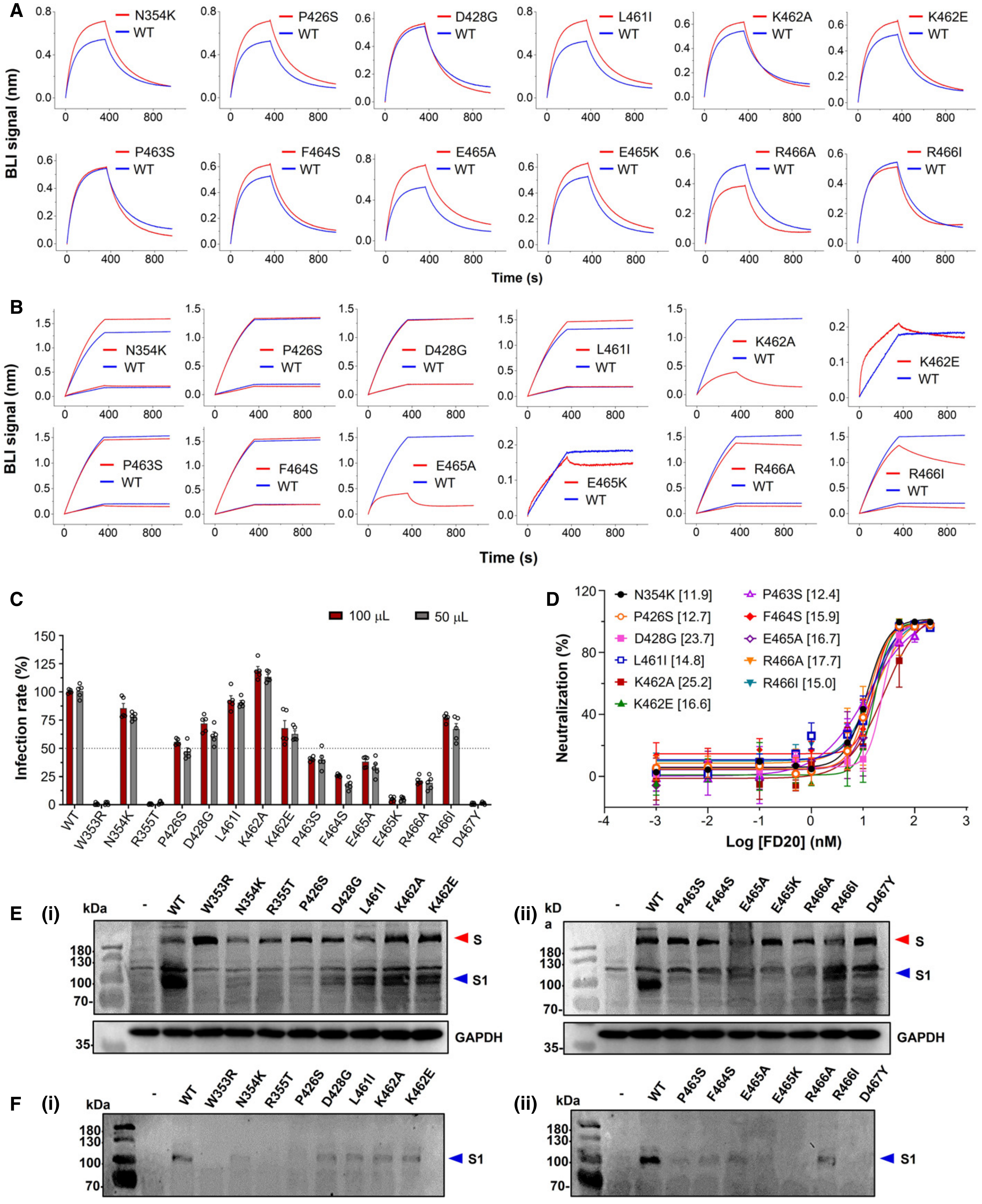

Figure EV2.

 © 2021 The Authors

Figure EV3. Mode of action of antibodies targeting RBM and measure of their IC_{50} in combination with FD20.

A Neutralization assay with S-wild-type SARS-CoV-2 pp (bearing S from hCoV-19/China/CAS-B001/2020) using FD20 in comparison to CB6, CV30, REGN10933, REGN10987, CR3022, and the inactive mutant FD20(Y112R). Mean ± SD are plotted ($n = 3$ biological replicates) except for Y112R (mean of $n = 2$ biological replicates). Numbers in brackets indicate IC_{50} values in nM.

B Neutralization assay with S D614G SARS-CoV-2 pp using FD20 in comparison to CB6, CV30, REGN10933, REGN10987, CR3022, and the inactive mutant FD20(Y112R). Average from two independent experiments are plotted except for CR3022 and FD20 (mean ± SD, $n = 3$ biological replicates). Numbers in brackets indicate IC_{50} values in nM.

C–E Mode of action of mAbs using SARS-CoV-2 pp and following the experimental procedure described in cartoons. (C) FD20 can act on a *post-binding* step contrary to CB6, CV30, REGN10933, REGN10987, and CR3022. VeroE6-hACE2 cells were incubated for 1 h at 4°C with SARS-CoV-2 pp, then unbound pp were removed by washing and cells were shift to 37°C for infection (1). mAbs were added during *binding* (2) or *post-binding* (3). Mean ± SD are plotted ($n = 3$ biological replicates). $P < 0.0001$ (****) unless specified otherwise (2-tailed, unpaired t-test). (D) The effect of pre-incubation on neutralizing activity. VeroE6-hACE2 cells were infected by SARS-CoV-2 pp directly (as a control) (1), or after (2) or during co-incubation of cells with mAbs (3). For pre-incubation conditions (4 and 5), SARS-CoV-2 pp and mAbs were first co-incubated for 1 h at 4°C (4) or 37°C (5), then the premix was used to infect VeroE6-hACE2 cells for 6 h at 37°C. Mean ± SD are plotted ($n = 3$ biological replicates). $P < 0.0001$ (****) unless specified otherwise (one-way ANOVA with Tukey's test). (E) The gain of neutralization activity by the pre-incubation step is more for FD20 than for control mAbs. Data are from (D). $P < 0.0001$ (****) unless specified otherwise (one-way ANOVA with Tukey's test). In (C) and (D), as control, cells were incubated at each step with an equal volume of PBS. Percentages of primary infection were calculated according to viral titers of PBS control conditions.

F Neutralization assay with SARS-CoV-2 pp using cocktails of FD20 with CB6, CV30, REGN10933, REGN10987, CR3022, and Y112R. 1:1 equimolar mix of FD20+mAbs were pre-incubated for 1 h at 4°C with SARS-CoV-2 pp, then the premix at different concentrations was incubated with VeroE6-hACE2 cells for 6 h at 37°C. The infection was analyzed by flow cytometry at 48 h post-infection. Concentration refers to the total molar concentration of mAbs. Numbers in brackets indicate IC_{50} values in nM. The slightly higher neutralizing activity of CV30+FD20 compared with CV30+Y112R indicates modest synergy of the two mAbs (Y112R is a null mutant of FD20). The same set of FD20+Y112R data are used for all panels. Data for FD20-containing cocktail (black) and FD20+Y112R (blue) are plotted as mean ± SD from three biological experiments except for FD20+CV30 ($n = 5$). Data for Y112R-containing cocktails (red) are plotted as mean from two independent experiments except for CV30+Y112R (mean ± SD, $n = 5$).

© 2021 The Authors

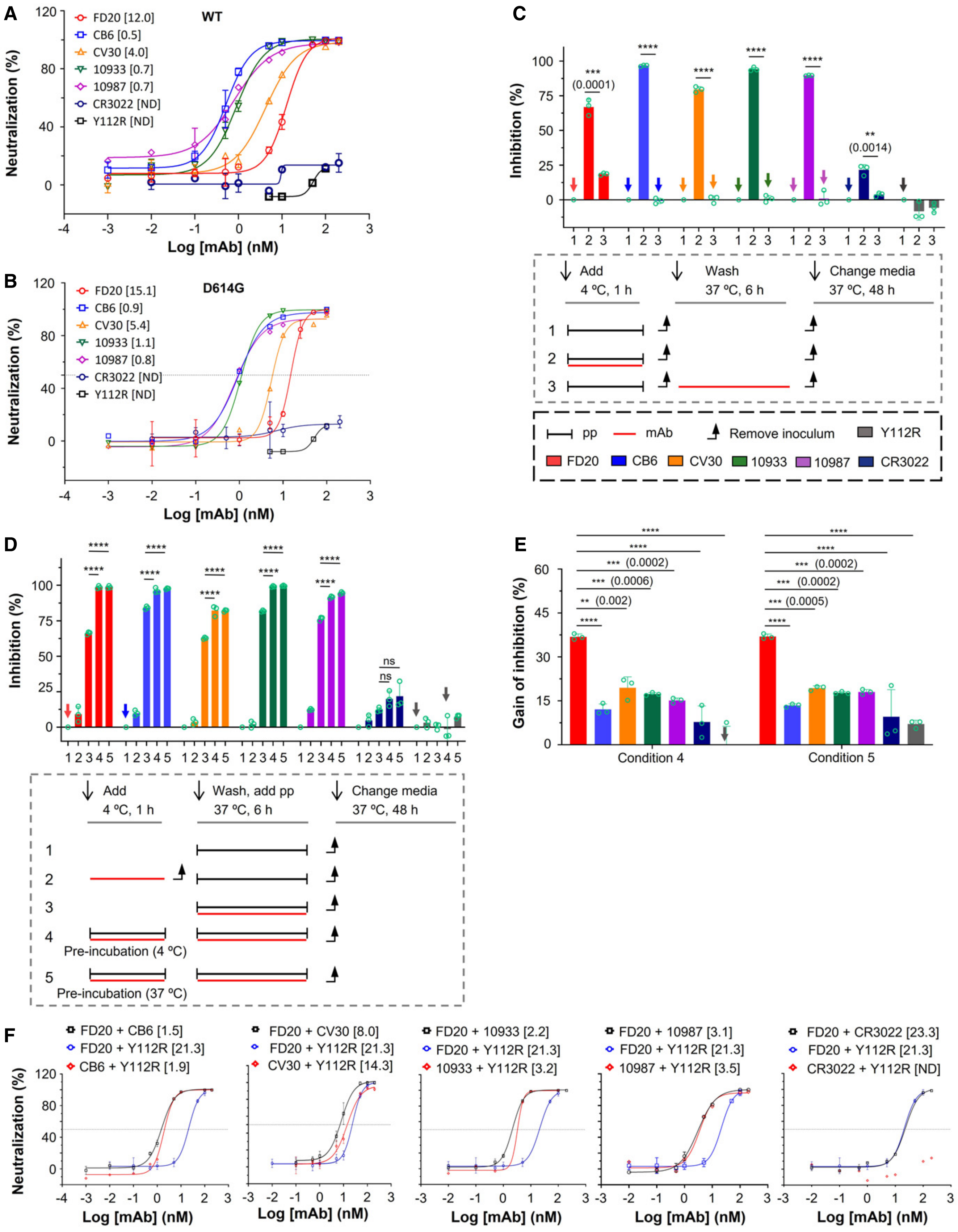

Figure EV3.

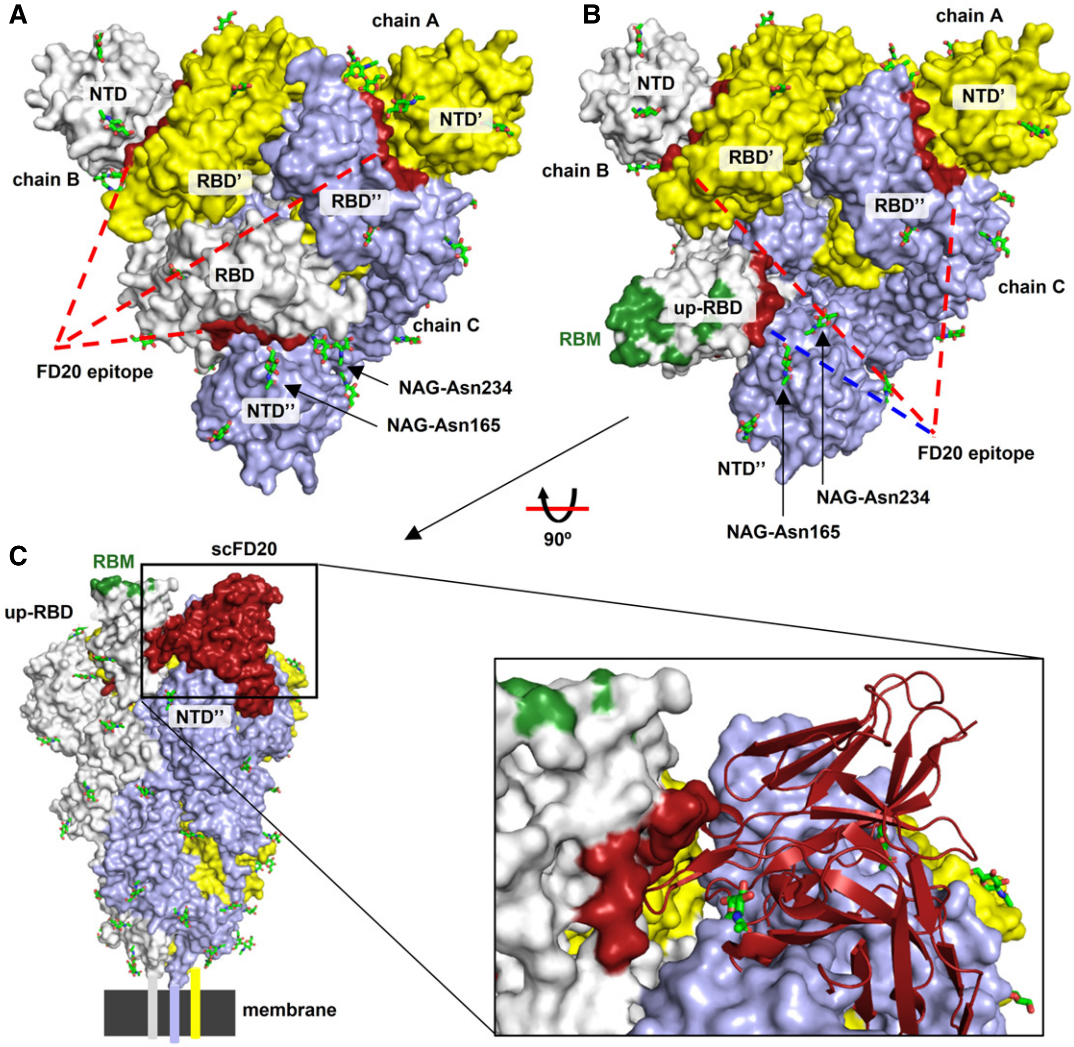

Figure EV4. FD20 binding is incompatible with the two pre-fusion conformations of SARS-CoV-2 S.

A The FD20 epitope in the context of the S trimer at the "closed" state (PDB ID 6vxx) (Walls *et al*, 2020). S subunits are shown as yellow, white, and light blue surfaces. Prime and double primer symbols distinguish domains from different subunits. The FD20 epitope (dark red) is buried in the narrow grove between the RBD and NTD from an adjacent monomer. Red lines indicate no accessibility, and a blue line indicates modest accessibility.

B The FD20 epitope in the context of the S trimer at the "open" state with one "up"-RBD (PDB ID 6vyb) (Walls *et al*, 2020). The FD20 epitope is marked dark red, and the receptor (ACE2)-binding site (RBM) on the "up"-RBD is marked green. Red lines indicate no accessibility, and a blue line indicates modest accessibility.

C Aligning the scFD20-RBD structure to the "up-"RBD reveals severe clashes between FD20 and the NTD from the anti-clock monomer. Schematic drawing at the bottom indicates the relative position of the membrane and the transmembrane helices.

Data information: A, B, top view (perpendicular to the membrane); C, side view. NAG, N-acetyl glucosamine. Glycans are shown as green sticks.

© 2021 The Authors

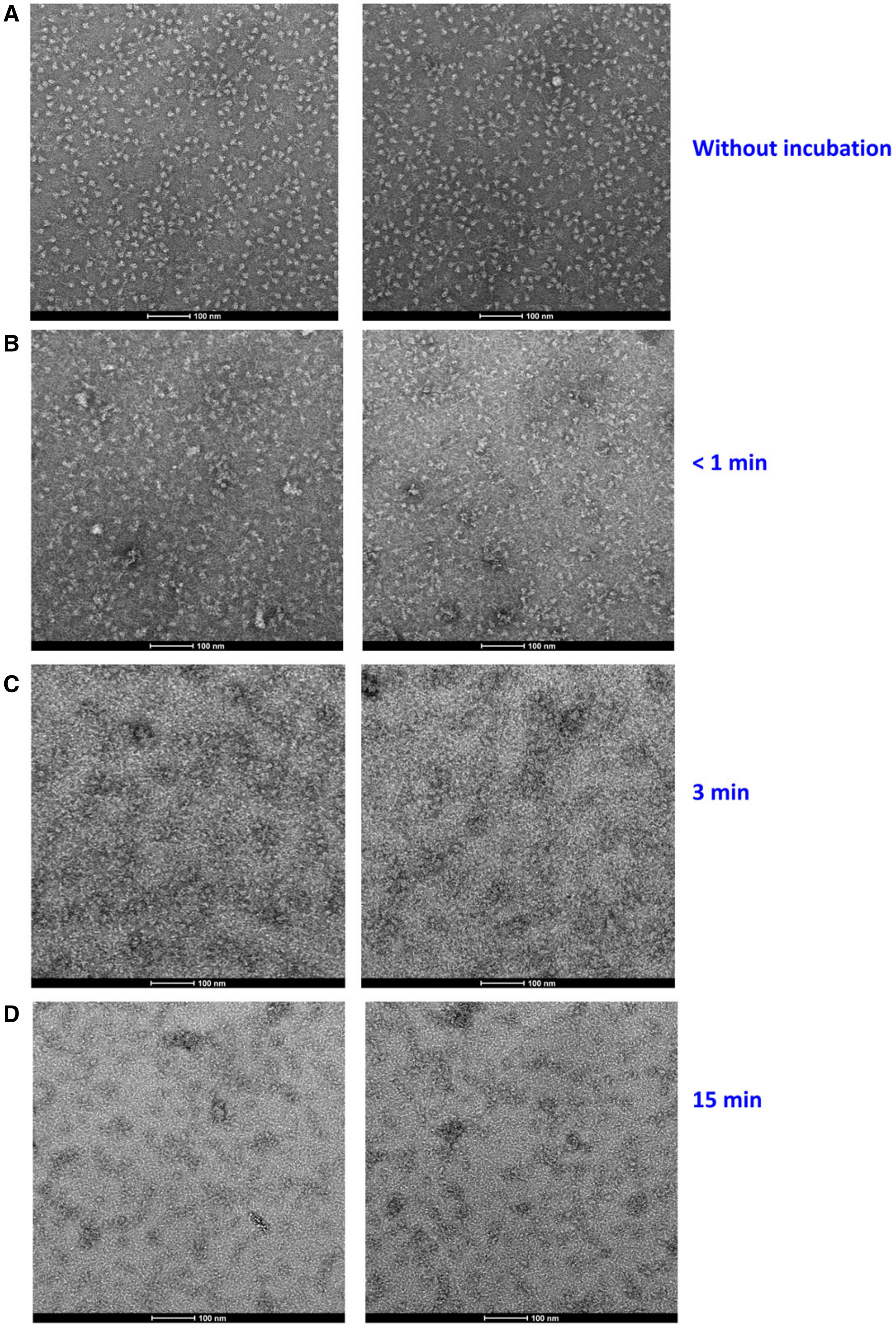

Figure EV5. FD20 destructs S-2P trimer within 3–15 min.

A Negative staining electron microscopy of S-2P (a stable mutant of S, see Methods) without FD20 incubation.

B–D Negative staining electron microscopy of S-2P immediately after the addition of FD20 (B), or after 3 min (C) or 15 min (D) of FD20 incubation. Bars are indicated in each panel. Two representative images are shown for each treatment.

 © 2021 The Authors
