# Supplementary figures and images for: Uncovering a conserved vulnerability site in SARS‐CoV‐2 by a human antibody
Source: EMBO Mol Med. 2021 Nov 17;13(12):e14544. doi: 10.15252/emmm.202114544 (PMC8646660; doi:10.15252/emmm.202114544)

**Fig EV2 E (i)**

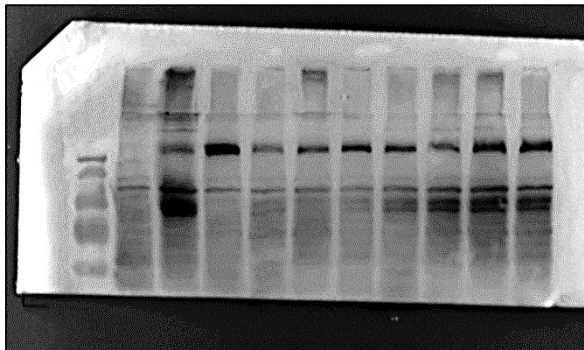

**Fig EV2 E (ii)**

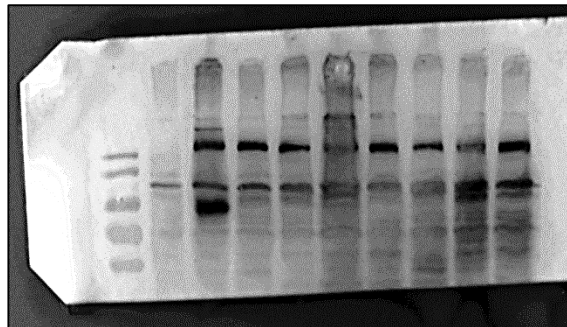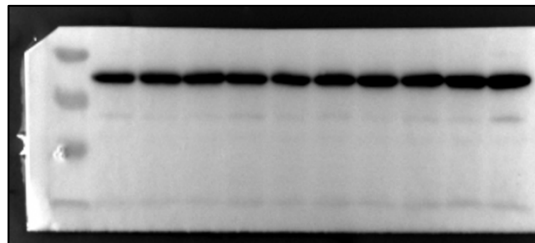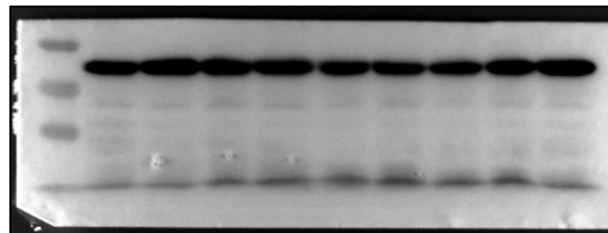

**Fig EV2 F (i)**

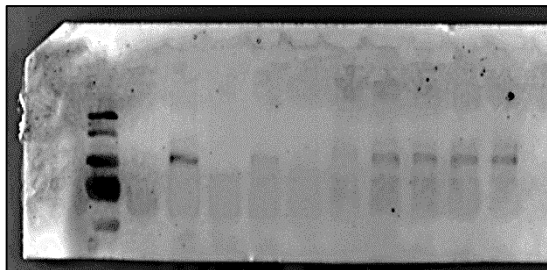

**Fig EV2 F (ii)**

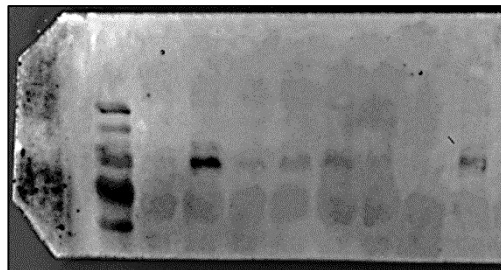

Supplement: Supplementary file 3 — Source Data for Expanded View [file EMMM-13-e14544-s007.zip › emmm202114544-sup-0009-SDataFigEV2EF.pdf]
